# A Comparison of the Incidence Rate for Joint Bleeding and FVIII Consumption between On-Demand versus Prophylaxis Factor VIII Replacement Therapy and the Direct Cost of Prophylaxis Treatment in Severe Haemophilia A Patients

**DOI:** 10.21315/mjms2023.30.1.11

**Published:** 2023-02-28

**Authors:** Munirah Jamal, Jameela Sathar, Jamaliah Jamari, Shamin Mohd Saffian

**Affiliations:** 1 Centre for Medicines Management, Faculty of Pharmacy, Universiti Kebangsaan Malaysia, Kuala Lumpur, Malaysia; 2 Department of Haematology, Ampang Hospital, Selangor, Malaysia; 3 Department of Pharmacy, Ampang Hospital, Selangor, Malaysia

**Keywords:** haemophilia, factor VIII, prophylaxis, bleeding rate, haemorrhage

## Abstract

**Background:**

Haemophilia A is a bleeding disorder caused by inadequate clotting factor VIII (FVIII). There are two main modes of treatment approach in severe haemophilia A patients either with on-demand or prophylaxis therapy with clotting factor FVIII concentrates. In this study, a comparison was made between the bleeding incidence rate of the on-demand and prophylaxis group in severe haemophilia A patients at Ampang Hospital, Malaysia.

**Methods:**

A retrospective study involving patients with severe haemophilia was conducted. The patient’s self-reported bleeding frequency was retrieved from the patient’s treatment folder from January to December 2019.

**Results:**

Fourteen patients received on-demand therapy, while the other 24 patients received prophylaxis treatment. The total number of joint bleeds in the prophylaxis group was significantly lower compared to the on-demand group (2.79 bleeds versus 21.36 bleeds [*P* < 0.001]). Furthermore, the total annual usage of FVIII was higher in the prophylaxis group compared to the on-demand group (1,506 IU/kg/year [± 905.98] versus 365.26 IU/kg/year [± 223.90], *P* = 0.001).

**Conclusion:**

Prophylaxis FVIII therapy is an effective treatment in reducing the frequency of bleeding joints. However, this treatment approach is associated with high cost due to the high consumption of FVIII.

## Introduction

Haemophilia A is a congenital, X-linked inherited bleeding disorder caused by a deficiency in clotting factor VIII (FVIII), where the patient is constantly at risk of bleeding. It can be classified as mild, moderate and severe: 5% to < 40%, 1%–5% and < 1% of the normal plasma FVIII level (1). Patients with severe haemophilia A can be treated with either on-demand therapy, which aims to stop the bleeding once it occurs or prophylaxis therapy by infusing FVIII regularly to prevent bleeding (1). On-demand therapy is more convenient and cost effective for patients due to the lower frequency of injections and FVIII consumption. However, the World Federation of Haemophilia (WFH) has recommended prophylaxis therapy as the standard of care for severe haemophilia patients (1). There are several limitations to the prophylactic use of FVIII, such as limited resources, difficulty in venous access and patient’s daily activity.

Despite the strong evidence on the effectiveness of prophylaxis treatment, this treatment approach remains costly because it requires a routine infusion of FVIII for twice to thrice weekly and a life-long commitment. In addition, the cost of treatment depends on the type of FVIII concentrates (human plasma-derived vs recombinant), the dosage of prophylaxis (low dose or high dose) and the development of inhibitors. Inhibitors are proteins that neutralise FVIII activity in the plasma caused by the high intensity of FVIII during the initial exposure (2, 3). In addition, some patients with inhibitors will respond to immune tolerance induction therapy, whereby a high concentration of FVIII was given regularly to induce antibody tolerance (4).

At Ampang Hospital, prophylaxis or on-demand therapy has been practised for severe haemophilia A patients since 2012. However, there is a lack of published data to compare the bleeding incidence rates between the treatment approaches. Therefore, this retrospective study aims to compare the joint bleeding incidence rate, FVIII consumption and direct cost and adherence of prophylaxis treatment compared to on-demand treatment in adult severe haemophilia A patients.

## Methods

### Study Design and Participants

This single-centre retrospective study involved patients with severe haemophilia A (defined as FVIII < 1% upon diagnosis) aged > 12 years old. The study was conducted at the haemophilia clinic in Ampang Hospital, Malaysia. Ampang Hospital is a tertiary hospital with a comprehensive Haemophilia Treatment Centre, which provides a full range of services necessary for patient management. Severe haemophilia patients without inhibitors must commit to a routine follow up at the Haemophilia Medication Therapy Adherence Clinic (HMTAC), attended by a multidisciplinary team that specialises in managing haemophilia.

All patients with severe haemophilia A (defined as FVIII < 1% upon diagnosis) aged 12 years old and above, receiving on-demand or prophylaxis therapy without a history of inhibitor in the past 12 months from the time of the study were included in this research. Meanwhile, patients with any bleeding disorders other than haemophilia A or who underwent surgical intervention in the past 12 months (January 2019–December 2019) were excluded from this study.

### Outcomes

At the Haemophilia Treatment Centre, a multidisciplinary team manages the haemophilia patients, consisting of haematologists, orthopaedic surgeons, pharmacists, physiotherapists and nurses. All interventions done by the team were recorded and compiled in a folder for each patient. The folder contains information related to the patient’s progress, bleeding log, infusion log and amount of FVIII supplied for each visit. Furthermore, the patients’ bleeding logs were reviewed and the following data were collected for analysis: demographics, comorbidities, target joints (bleeding occurs at the same joint ≥ 3 times within the past 6 months), history of severe bleeding (intracranial, throat, gastrointestinal and iliopsoas bleeding), mode of treatment (on-demand or prophylaxis), FVIII dose per infusion, number of joint bleeding over 12 months and total IU of FVIII used over 12 months.

### Other Outcomes Measured

The adherence level of patients on prophylaxis treatment was measured using the stock reconciliation method. The calculation was made based on the patient’s actual number of prophylaxis infusions divided by the total prophylaxis infusion calculated in a year (4). In this study, the patient is considered adherent if more than 80% FVIII was administered.

### Statistical Methods

The data collected were analysed using the Statistical Package for Social Sciences (SPSS) version 25.0. Descriptive statistics were determined using the demographic data, prescribed dose of FVIII, number and site of target joint and total IU of FVIII used per year. Meanwhile, categorical data such as race, body mass index (BMI), comorbidities, history of severe bleeding, type of treatment and site of target joint were presented as frequency and percentage. Continuous data such as age, weight, height, prescribed dose of FVIII, number of joint bleeding and total IU of FVIII used in a year were presented as mean ± standard deviation.

The outcome on the bleeding incidence rate (defined as the total number of bleeding events in a year) and total FVIII used in a year between the on-demand and prophylaxis groups was compared using the independent t-test. In addition, potential variables such as prescribed FVIII dose, BMI, adherence to the prescribed prophylaxis regiment, were analysed to explore the significant factors associated with the bleeding incidence rate. All statistical tests with *P* < 0.05 denote statistical significance.

## Results

### Demographic Characteristics

Thirty-eight adults with severe haemophilia A patients were included in this study. Generally, the demographic and characteristics of the patients between the prophylaxis and on-demand groups were comparable (Table 1). The patients’ mean age was 36 ± 15.5 years old and 43.57 ± 13.3 years old in the prophylaxis and on-demand group, respectively. All major ethnic groups in Malaysia were involved in this study. Chinese (*n* = 17, 44.7%), Malay (*n* = 16, 42.1%) and Indian (*n* = 5, 13.2%). The ethnicities between prophylaxis and on-demand groups were not statistically significant (*P* = 0.830).

The patients’ mean weight were 62.87 (± 13.7) kg and 66.64 (± 10.0) kg in the prophylaxis and on-demand groups, respectively. However, there were more obese patients in the prophylaxis group (5/24, 20.8%) compared to the on-demand group (1/14, 7.1%) but their BMI were not statistically different (*P* = 0.227).

### Outcome Measures

Almost all patients had at least one target joint (unilateral or bilateral joint) bleeding reported upon the first treatment visit to the centre, but there was no significance between the two groups. The affected joints were as follows: ankle joint (25 patients), followed by elbow joint (23 patients) and knee joint (14 patients). Furthermore, 28.6% of patients from the on-demand group had a history of intracranial bleeding compared to the prophylaxis group (4.2%) (Table 1) but the difference was not statistically significant (*P* = 0.158).

The average joint bleeding incidence rate and site of joint bleeding are shown in Table 2. Overall, the joint bleeding occurrence was significantly lower (*P* < 0.001) in the prophylaxis group 2.79 (± 3.51) compared to the on-demand group, 21.36 (± 13.33). In this study, three types of joint bleeding were analysed: elbow, knee and ankle. On-demand group recorded more bleeding at elbow joint (9 [64.3%] versus 8 [33.3%]; *P* = 0.032) and ankle joint (10 [71.4%] versus 8 [33.3%]; *P* = 0.012) compared to the prophylaxis group. However, prophylaxis group reported higher cases in knee bleeding (8 [33.3%] versus (5 [35.7%]; *P* = 0.441).

The unit of FVIII used was slightly higher in the prophylaxis group than the on-demand group (16.35 ± 2.55 IU/kg versus 17.14 ± 2.16 IU/kg; *P* = 0.170). Additionally, the annual total FVIII usage was higher in the prophylaxis group than the on-demand group (1506.03 ± 905.98 IU/kg/year versus 365.26 ± 223.90 IU/kg/year; *P* = 0.001). When the cost of treatments was compared, the prophylaxis group recorded significantly higher total treatment cost than the on-demand group (RM786.54 [456.92; 1103.25] IU/kg/year versus RM166.68 [132.90; 216.57] IU/kg/year). Meanwhile, 87.5% of patients in the prophylaxis group adhered to the treatment regime.

## Discussion

This retrospective study analysed the bleeding incidence rates of 38 patients with severe haemophilia A in Malaysia. The joint bleeding incidence rate was lower in the prophylaxis group than the on-demand group. Based on the drug expenditure report in 2019, approximately RM3 million were spent on clotting factors to treat approximately 150 patients with haemophilia A (data on file) in the first half of 2019. Despite the small sample size (38 patients) used in the present study, the findings offer important information on the current status of haemophilia treatment in Malaysia, which is useful for planning future research.

In the prophylaxis group, FVIII was infused twice or thrice weekly to prevent the spontaneous bleeding event, a common presentation in severe haemophilia A patients. Regular infusion of FVIII is a strategy to target the trough FVIII level of more than 1%. Thus, severe haemophilia A patients will have none or fewer spontaneous bleeding events like those with moderate and mild haemophilia A. In addition, the current findings aligned with previous studies conducted in developing countries (5–11).

Bleeding into the joints remains a common complication of haemophilia. When the joint bleeding occurs in the same joint more than three times within a consecutive 6-month period, the joint will be known as a target joint. This study has found that most patients had at least one target joint, with the ankle being the most common target joint (25 out of 38 patients), followed by the elbow (23 out of 38 patients) and the knee (14 out of 38 patients). Similarly, an earlier study reported the same target joint pattern (10). However, some differences were evident such as the ankle joint being the most common joint affected among severe haemophilia A patients instead of the knee joint. Furthermore, the knee was the most affected joint when assessed using the joint function clinical scores. On the contrary, the ankle was the most commonly affected joint in severe haemophilia A patients when assessed using Patterson radiological scoring. The findings suggest that radiological scoring is the preferred scoring tool in describing underlying joint changes in the ankle. Another mechanism that might contribute to the ankle being the most affected joint in severe haemophilia A patient was the introduction of prophylaxis therapy and the availability of effective treatment at home. This treatment option allows patients to be involved in high impact sports and activities, thus increasing their physical abilities. The ankle joint is subjected to greater weight-bearing since it is the first to absorb the forces of weightbearing and body motion, thus, increasing bleeding incidence (5).

Data comparing tertiary prophylaxis with on-demand treatment are limited. Most studies have a methodological limitation that includes retrospective or non-controlled studies, varying dose regimen used, and a limited sample size. In the current study, patients in the prophylaxis group began treatment either after two large joint bleeding events (secondary prophylaxis) or after developing haemophilic arthropathy (tertiary prophylaxis) [data on file]. On the other hand, regular prophylaxis with FVIII starts later, after patients are presented with target joints in adult haemophilia A. This treatment option is not intended to reverse the joint disability, but to delay the progression of joint disease by reducing the number of bleeding episodes to improve the patient’s quality of life.

In the study of Prophylaxis versus On-demand Therapy Through Economic Report POTTER (7), late prophylaxis in adults had shown a significantly reduced joint bleeding frequency compared to the on-demand group. A 5-years follow up demonstrated a beneficial effect on joint outcomes in the prophylaxis group, particularly in older patients. Besides, prophylaxis may delay the progression of arthropathy based on the Patterson score. The positive outcome on the joint function in an adult with late prophylaxis treatment has been confirmed even after 3 years of prophylaxis treatment, as reported in the prospective randomised trial of Secondary Prophylaxis with recombinant FVIII Therapy in Severe Hemophilia A Adult and/or Adolescent Subjects Compared to that of Episodic Treatment (SPINART) (6).

The current average dose of FVIII (16.35 ± 2.55) IU/kg was classified as low dose prophylaxis, which falls within the recommended dose as proposed by the Utrecht protocol (15 IU/kg–30 IU/kg) (12). Moreover, a similar average dose used in on-demand and prophylaxis groups resulted in a significantly reduced number of annual joint bleeding for an adult patient with severe haemophilia A. This outcome aligned with other studies conducted in China, Thailand, Tunisia, India and Indonesia (9, 13–15). The average dose used in their study was 8 IU/kg–15 IU/kg, administered twice or thrice weekly. On top of that, these studies reported improvements in patients’ joint health using different scoring.

The present findings also agreed with Wong et al. (16), where the prophylaxis treatment group exhibited four times higher total annual FVIII usage than the on-demand group due to the regular FVIII infusion. Besides, there were more overweight and obese patients in the prophylaxis group than the on-demand group, which might increase the FVIII usage due to weight-based dosing. Furthermore, overweight and obesity among haemophilia patients were associated with clinically significant complications, including musculoskeletal disease, aerobic capacity, cardiovascular disease, diabetes, hyperlipidaemia, decreased quality of life and change in the pharmacokinetics of infused clotting factor (16). Meanwhile, the global prevalence of overweight and obesity in haemophilia was 17% (4). This information suggests the need for weight management strategies for haemophilia patients.

The present study also evaluated adherence among patients in the prophylaxis group and found that 87.5% adhered to their treatment regimen. Several methods can be used to assess adherence among haemophilia patients, including patient-reported data from treatment logs, pharmacy records, bleeding logs and sports/physical activity (4). The adherence level in this study was determined based on the data from the treatment logs review and pharmacy records. Patients were considered adherent to the treatment if they infused more than 80% of FVIII as prescribed by the physician. Nevertheless, using a single method in assessing adherence level is subjected to inherent bias and confounding factors. Thus, a combination of adherence assessment methods should be conducted in the future to ensure accurate adherence level determination.

Patients with severe haemophilia A require lifelong of FVIII replacement therapy. The main challenges in practising prophylaxis are related to the high cost of this therapy. Clotting factors contribute about 90% of direct cost of treatment in haemophilia. The current findings showed that the annual direct cost of prophylaxis therapy per patient was about four times higher compared to on-demand therapy. Despite the high cost of therapy, prophylaxis is a cost-effective strategy, as demonstrated by the quality-adjusted life-year (QALY) values (17, 18). It should be noted that the reported economic evaluation studies were done mainly in European countries that evaluate cost-effectiveness of primary prophylaxis in a paediatric population.

From healthcare and societal perspectives, an Italian study by Abbonizio et al. (18) revealed late prophylaxis to be more cost-effective than on-demand therapy where incremental cost-effectiveness ratios (ICERs) equal €53,978/QALY, which falls below the acceptable threshold in Italy. The cost-effectiveness study outcomes might differ from one country to another since it is sensitive to several factors, including the cost of FVIII, varying frequency and dose regimen, and ICER threshold value.

According to the WFH guideline (19), prophylaxis therapy is still a reasonable approach in countries with limited resources using a low dose ‘Utrecht’ regimen (12) to treat patients with severe haemophilia A, as demonstrated by earlier studies with positive outcomes (8, 12–15).

Several limitations have been identified in the present study. Firstly, the longitudinal data analysis was not carried out due to the relatively short follow-up period of 12 months, which would have provided a better picture of the changing outcomes with time. Secondly, the number of samples is small since this is a single centre study and haemophilia is a relatively rare disease. However, it is also important to note that this is the main haemophilia treatment centre for adult patients in Malaysia. Furthermore, this study did not represent several populations, such as obese or geriatric patients, due to the small sample size. In addition, paediatric patients and patients with moderate and mild haemophilia were excluded due to the absence of readily available data records, preventing the results from generalising these populations. Another limitation is the large variance compared to the average bleeding incidence rates. This indicates that there are a lot of variability which has not been explained in our current dataset and therefore further studies with large sample size are needed to explain this variance. Lastly, the univariate analysis did not consider other factors influencing the joint bleeding incidence rate among haemophilia A patients, such as body weight, physical activities and occupation.

### Conclusion

Prophylaxis treatment in adult with severe haemophilia A resulted in a lower joint bleeding incidence rate than the on-demand treatment approach (2.79 bleeds versus 21.36 bleeds; *P* < 0.01). However, prophylaxis treatment is associated with approximately 4.7 times higher in FVIII usage than on-demand therapy, with estimated cost of RM786 (457; 1103) IU/kg/year versus RM167 (133; 217) IU/kg/year, respectively.

## Figures and Tables

**Table 1:** Demographics and patient characteristics

Characteristics	Prophylaxis group *n* = 24	On-demand group *n* = 14	*P*-value
Age, mean (SD)	36.00 ± 15.5	43.57 ± 13.3	0.136
Ethnicity, *n* (%)			0.830
Malay	11 (45.8)	5 (27.8)	
Chinese	10 (41.6)	7 (50)	
Indian	3 (12.5)	2 (14.3)	
Weight, mean (SD)	62.87 ± 13.7	66.64 ± 10.0	0.634
BMI			0.227
Underweight	3 (4.2)	0	
Normal weight	10 (41.7)	10 (71.4)	
Overweight	16 (66.7)	3 (21.4)	
Obesity	5 (20.8)	1 (7.1)	
Target joint, *n* (%)			0.082
None	1 (4.2)	0 (0)	
< 1 target joint	17 (70.8)	14 (100)	
> 1 target joint	6 (25)	0 (0)	
Site of target joint, *n* (%)			
Elbow			
None	10 (41.7)	5 (35.7)	0.247
Left or Right	8 (33.3)	8 (57.1)	
Both	6 (25.0)	1 (7.1)	
Knee			0.897
None	15 (62.5)	9 (64.3)	
Left or Right	8 (33.3)	4 (28.6)	
Both	1 (4.2)	1 (7.1)	
Ankle			0.245
None	8 (33.3)	5 (35.7)	
Left or right	9 (37.5)	8 (57.1)	
Both	7 (29.2)	1 (7.1)	
History of serious bleed, *n* (%)			0.158
None	17 (70.8)	7 (50)	
Throat	0 (0)	0 (0)	
Iliopsoas	5 (20.8)	3 (21.4)	
Gastrointestinal	0 (0)	0 (0)	
Intracranial bleeding	1 (4.2)	4 (28.6)	

Note: SD = standard deviation

**Table 2:** Joint bleeding rates and site of joint bleeding

Variables	Prophylaxis group (*n* = 24)	On-demand group (*n* = 14)	*P*-value

Average joint bleeding events in a year, Mean (SD)	2.79 ± 3.514	21.36 ± 13.33	0.001
Site of joint bleeding, *n* (%)			
Elbow	8 (33.3)	9 (64.3)	0.032
Knee	8 (33.3)	5 (35.7)	0.441
Ankle	8 (33.3)	10 (71.4)	0.012
